# Incidence and Risk Factors for Hospital-Acquired Pressure Ulcers in Patients With COVID-19

**DOI:** 10.7759/cureus.80113

**Published:** 2025-03-05

**Authors:** Hideharu Nakamura, Takaya Makiguchi, Yumi Yamada, Aya Tsunoda, Nana Tomaru, Satoshi Yokoo

**Affiliations:** 1 Department of Plastic and Reconstructive Surgery, National Hospital Organization (NHO) Takasaki General Medical Center, Takasaki, JPN; 2 Department of Oral and Maxillofacial Surgery, and Plastic Surgery, Gunma University Graduate School of Medicine, Maebashi, JPN

**Keywords:** covid-19, hospital-acquired pressure ulcer, independence in activities of daily living, isolation, multidisciplinary care

## Abstract

Background: Hospital-acquired pressure ulcers (HAPU) are a major challenge in healthcare settings, especially in vulnerable populations such as patients with COVID-19. The aim of this study is to examine the association of COVID-19 infection with occurrence of HAPU, with the goal of enhancing care protocols and reducing HAPU incidence in at-risk populations.

Methods: A retrospective analysis was conducted for 8,158 hospitalized patients aged 20 years and older who were treated from April 2022 to March 2023. The study included COVID-19-positive patients and non-COVID-19 patients. Data were collected for age, sex, body mass index, total protein, albumin (ALB), hemoglobin, COVID-19 infection, and independence in activities of daily living (ADL). For patients with COVID-19, surgical history, intensive care unit admission, physical restraints, mechanical ventilation, and prone positioning therapy were also examined. Risk factors for HAPU were evaluated based on these variables.

Result: In all 8,158 patients, low ALB and COVID-19 infection were independent risk factors for HAPU. In the COVID-19 cohort (n=412), low independence in ADL was also an independent risk factor. These findings underscore the importance of targeted interventions to mitigate the occurrence of HAPU, particularly for high-risk COVID-19 patients.

Conclusion: This study showed that advanced age, low ALB, and COVID-19 infection are significant independent risk factors for HAPU. For COVID-19 patients, isolation policies may have reduced caregiver-patient interactions and hindered the delivery of frequent care, thereby increasing the risk of HAPU, particularly in patients with low ADL independence. For high-risk patients, a multidisciplinary approach is essential, with tailored PU prevention strategies implemented early to address individual needs and reduce the risk of HAPU.

## Introduction

Hospital-acquired pressure ulcer (HAPU) is defined as a pressure ulcer (PU) that develops during hospitalization. This condition is a critical concern in healthcare settings. Key risk factors include advanced age, limited mobility, poor skin condition, malnutrition, and use of medical devices [1]. HAPU is associated with prolonged hospital stays [2] and increased medical costs [3], which place an additional burden on patients. Thus, the need for ongoing prevention and management of these complications is paramount.

The COVID-19 pandemic increased the risk of development of HAPU. Patients with COVID-19 often have endothelial cell damage and hypercoagulability, which increase their risk of microvascular injury and reduce skin tolerance to pressure [4]. The unique challenges of isolation protocols for patients with COVID-19, including restricted patient mobility, shifts in care priorities, and the impact of personal protective equipment, further exacerbate these risks [5]. Therefore, there is a need to identify specific risk factors for HAPU in patients with COVID-19 and to develop targeted prevention strategies.

The aims of this study are to examine the relationship of COVID-19 infection with occurrence of HAPU and identify independent risk factors for HAPU. By examining data from hospitalized patients in a controlled setting, we seek to contribute to improved care protocols and reduce the incidence of HAPU in this vulnerable population.

## Materials and methods

Study design and patient population

A retrospective observational study was conducted at a single tertiary care hospital. A retrospective analysis was performed on 8,158 hospitalized patients aged 20 years and older who received treatment between April 2022 and March 2023. Patients hospitalized for at least two days who underwent blood tests for total protein, albumin, and hemoglobin were included in the study. To ensure data completeness and accuracy, patients with missing data for albumin levels, BMI, or independence in ADL were excluded. Additionally, patients with pre-existing pressure ulcers at the time of admission were excluded. Data were extracted from the hospital’s electronic medical records system using a standardized data collection protocol to maintain accuracy and consistency. The study included COVID-19-positive patients and non-COVID-19 patients. Variables analyzed included age, sex, body mass index (BMI), total protein (TP), albumin (ALB), hemoglobin, COVID-19 infection, and independence in activities of daily living (ADL). For patients with COVID-19, surgical history, intensive care unit (ICU) admission, physical restraints, mechanical ventilation, and prone positioning therapy were also evaluated. Independence in ADL was assessed using a standardized classification: fully independent (Rank 1), semi-bedridden (Rank 2), bedridden but able to maintain a sitting position with assistance (Rank 3), and completely bedridden (Rank 4) [6]. The final dataset comprised 8,159 patients in the whole cohort and 413 patients in the COVID-19 cohort.

Statistical analysis

All patient data were anonymized before analysis. Statistical analysis continuous variables were tested for normality using a Kolmogorov-Smirnov test. Normally distributed variables were compared using t-tests, while non-normally distributed variables were analyzed by Mann-Whitney U test as a non-parametric alternative. Categorical variables were evaluated by Fisher exact test to identify potential risk factors associated with HAPU. Variables with significant associations (p < 0.05) in univariate analysis were included in multivariate logistic regression models to determine independent risk factors for HAPU. Data analyses were conducted using EZR (Saitama Medical Center, Jichi Medical University, Saitama, Japan), a graphical user interface for R (The R Foundation for Statistical Computing, Vienna, Austria, v. 4.3.1). EZR is a modified version of R Commander (v. 2.9-1) with additional functions for biostatistics [7].

Ethical considerations

This study was conducted in accordance with the principles outlined in the Helsinki Declaration and received approval from the research ethics committee of the National Hospital Organization (NHO) Takasaki General Medical Center (No. TGMC2024-073). Due to the retrospective nature of the study, the requirement for individual patient consent was waived. 

## Results

Whole cohort

In all 8,158 patients, the incidence of HAPU was significantly associated with advanced age (p < 0.001), lower BMI (p < 0.001), lower TP (p < 0.001), lower ALB (p < 0.001), lower Hb (p <0.001), and COVID-19 infection status (p = 0.00192) (Table 1). Multivariate logistic regression identified advanced age (p = 0.0353), lower ALB (p < 0.001), and COVID-19 infection (p = 0.0270) as independent risk factors for HAPU (Table 2).

**Table 1:** Univariate Analysis of Factors Associated with HAPU Data are presented as mean ± standard deviation (SD) for continuous variables and as counts and percentages for categorical variables. HAPU: Hospital-acquired pressure ulcer. *P<0.05, **P<0.01, P<0.001***

Parameter		No HAPU (n=8,030)	HAPU (n=128)	P-value
Age	(years)	68.7 ± 15.8	75.3 ± 13.1	< 0.001***
Sex	(M/F)	4628/3402 (57.6% Male)	75/53 (58.6% Male)	0.857
Body mass index	(kg/m2)	22.7 ± 4.2	21.2 ± 5.1	< 0.001***
Total protein	(g/dL)	6.64 ± 0.76	6.04 ± 0.96	< 0.001***
Albumin	(g/dL)	3.74 ± 0.69	3.05± 0.78	< 0.001***
Hemoglobin	(g/dL)	12.68 ± 2.24	11.44 ± 2.49	< 0.001***
Independence in activities of daily living	(1-4)	1.92 ± 1.21	1.93 ± 1.24	0.858
COVID-19 infection	(Yes/No)	397/7633 (4.94% Yes)	15/113 (11.72% Yes)	0.00192**

**Table 2:** Multivariable Logistic Regression Analysis of Factors Associated with HAPU HAPU: Hospital-acquired pressure ulcer. *P<0.05, **P<0.01, P<0.001***

Parameter	Odds Ratio (OR)	95% Confidence Interval (CI)	P-value
Age	1.010	1.000 - 1.030	0.0353*
Body mass index	0.963	0.920 - 1.010	0.0986
Total protein	0.873	0.677 - 1.130	0.2950
Albumin	0.389	0.280 - 0.541	< 0.001***
Hemoglobin	1.000	0.914 - 1.100	0.9860
COVID-19 infection	1.880	1.070 - 3.300	0.0270*

COVID-19 cohort

In the COVID-19 cohort of 412 patients, univariate analysis revealed significant associations between HAPU and male sex (p = 0.00671), lower BMI (p = 0.0261), lower ALB (p = 0.0381, low independence in ADL (p < 0.001), and use of mechanical ventilation (p = 0.0106) (Table 3). Multivariate logistic regression identified low independence in ADL (p = 0.00459) as an independent risk factor (Table 4).

**Table 3:** Univariate Analysis of Factors Associated with HAPU in Patients with COVID-19 Data are presented as mean ± standard deviation (SD) for continuous variables and as counts and percentages for categorical variables. HAPU: Hospital-acquired pressure ulcer. *P<0.05, **P<0.01, P<0.001***

Parameter		No HAPU (n=397)	HAPU (n=15)	P-value
Age	(years)	63.3 ± 22.7	75.8 ± 14.3	0.057
Sex	(M/F)	172/225 (43.3% Male)	12/3 (80.0% Male)	0.00671**
Body mass index	(kg/m2)	22.1 ± 4.4	19.7 ± 4.6	0.0261*
Total protein	(g/dL)	6.37 ± 0.79	6.15 ± 0.60	0.278
Albumin	(g/dL)	3.40 ± 0.69	3.03± 0.53	0.0381*
Hemoglobin	(g/dL)	11.96 ± 2.14	11.74 ± 1.96	0.697
Independence in activities of daily living	(1-4)	1.44 ± 0.97	2.60 ± 1.55	< 0.001***
Surgical history	(Yes/No)	397/7633 (9.85% Yes)	15/113 (11.72% Yes)	0.955
Intensive care unit admission	(Yes/No)	68.7 ± 15.8	75.3 ± 13.1	< 0.001***
Physical restraint	(Yes/No)	70/327 (17.6% Yes)	4/11 (26.7% Yes)	0.323
Mechanical ventilation	(Yes/No)	22/375 (5.87% Yes)	4/11 (26.7% Yes)	0.0106*
Prone position therapy	(Yes/No)	2/395 (0.50% Yes)	1/14 (6.67% Yes)	0.106

**Table 4:** Multivariable Logistic Regression Analysis of Factors Associated with HAPU in Patients with COVID-19 HAPU: Hospital-acquired pressure ulcer. *P<0.05, **P<0.01, P<0.001***

Parameter	Odds Ratio (OR)	95% Confidence Interval (CI)	P-value
Sex	3.620	0.960 - 13.60	0.05750
Body mass index	0.859	0.733 – 1.010	0.06100
Albumin	0.627	0.291 - 1.350	0.23300
Independence in activities of daily living	1.780	1.200 - 2.660	0.00459**
Mechanical ventilation	3.880	0.978 – 15.40	0.05380

These findings highlight the importance of targeted interventions to mitigate the occurrence of HAPU, particularly for high-risk COVID-19 patients.

## Discussion

HAPU has a significant impact on patient outcomes and healthcare costs, leading to prolonged hospital stays [2], increased medical expenses [8], and higher mortality among critically ill patients [9]. Reported risk factors include advanced age, limited mobility, poor skin condition, incontinence, and malnutrition [10]. In this study, advanced age and low ALB were confirmed as independent risk factors for HAPU, consistent with previous reports. Older age is a significant risk factor for PU development in various healthcare settings [11], and malnutrition is another critical risk factor, especially in elderly patients [12]. Low serum ALB and TP are strongly correlated with the risk of PU [13,14], and zinc has also been identified as a nutritional marker linked to PU risk. In elderly people, zinc deficiency may be prevalent due to factors such as altered intestinal absorption, inadequate mastication, psychosocial factors, drug interactions, and altered subcellular processes [15], and zinc deficiency can exacerbate the severity of PU and delay healing [16]. Thus. improvement of nutritional status in older adults through proper assessment, adequate dietary intake, and addressing issues such as impaired cognition and swallowing difficulties can help to prevent and manage PU [17,18].

COVID-19 infection was also identified as an independent risk factor for HAPU. In patients with COVID-19, the incidence of HAPU ranges from 7.3% to 77.0%, with higher rates associated with prone positioning therapy and use of medical devices [19]. These patients often have endothelial cell damage and hypercoagulability, which predisposes them to microvascular injury and reduces their tolerance to pressure or shear forces on the skin and subcutaneous tissues [4]. Additionally, during the COVID-19 pandemic, increased medical staff turnover and reliance on traveling nurses have been reported to contribute to suboptimal care outcomes, including higher rates of hospital-acquired infections and prolonged hospital stays due to elevated workloads and insufficient training [20-22]. These factors likely had a detrimental impact on HAPU incidence as well, further emphasizing the need for adequate staffing, structured training programs, and enhanced wound care protocols to mitigate these risks.

In this study, independence in ADL was not a significant risk factor for HAPU in the total hospitalized population. However, low independence in ADL emerged as a significant independent risk factor for HAPU in patients with COVID-19, highlighting the increased vulnerability of this population. This finding suggests that isolation policies during the COVID-19 pandemic, particularly for patients with low independence in ADL, may have reduced caregiver-patient interaction and hindered the delivery of frequent care, such as repositioning and skin assessments, thereby increasing the risk of HAPU. Particularly in wound healing, early intervention has been reported as essential in regions where the number of healthcare providers is scarce relative to the patient population [23]. In such areas, limited caregiver-patient interaction may further exacerbate patient outcomes, potentially leading to worse prognoses.

A 6.2% incidence of HAPU within two days of hospitalization has been reported in high-risk older patients [24], emphasizing the importance of early risk assessment and preventive measures. High-risk patients require a multidisciplinary approach involving physicians, nurses, dietitians, and physical therapists, while comprehensive care plans should include routine assessments of nutritional status, skin condition, and mobility. Additionally, it is essential to develop and implement tailored HAPU prevention strategies that address the needs of each patient at an early stage. Regular communication among care team members about patient conditions and care progress is also vital for ensuring timely interventions.

The limitations of the study include its retrospective and single-center design. Future research should explore the findings in broader contexts, incorporating multicenter data and longitudinal design to validate and expand upon the results.

## Conclusions

Advanced age, low ALB, and COVID-19 infection were identified as significant independent risk factors for HAPU. Among patients with COVID-19, the additional risks associated with reduced independence in ADL highlight the heightened vulnerability of this population. In these patients, isolation policies may have reduced caregiver-patient interactions and hindered delivery of frequent care, such as repositioning and skin assessments, thereby increasing the risk of HAPU, particularly in patients with low ADL independence. For high-risk patients, a multidisciplinary approach is required through involvement of healthcare professionals such as physicians, nurses, dietitians, and physical therapists. It is also crucial to develop and implement tailored HAPU prevention strategies that address the specific needs of each patient at an early stage to mitigate risks.
